# Mapping brain asymmetry in health and disease through the ENIGMA consortium

**DOI:** 10.1002/hbm.25033

**Published:** 2020-05-18

**Authors:** Xiang‐Zhen Kong, Merel C. Postema, Tulio Guadalupe, Carolien de Kovel, Premika S. W. Boedhoe, Martine Hoogman, Samuel R. Mathias, Daan van Rooij, Dick Schijven, David C. Glahn, Sarah E. Medland, Neda Jahanshad, Sophia I. Thomopoulos, Jessica A. Turner, Jan Buitelaar, Theo G. M. van Erp, Barbara Franke, Simon E. Fisher, Odile A. van den Heuvel, Lianne Schmaal, Paul M. Thompson, Clyde Francks

**Affiliations:** ^1^ Language and Genetics Department Max Planck Institute for Psycholinguistics Nijmegen The Netherlands; ^2^ Department of Psychiatry, Amsterdam Neuroscience Amsterdam University Medical Center, Vrije Universiteit Amsterdam Amsterdam The Netherlands; ^3^ Department of Anatomy and Neurosciences, Amsterdam Neuroscience, Amsterdam University Medical Center Vrije Universiteit Amsterdam Amsterdam The Netherlands; ^4^ Department of Human Genetics Radboud University Medical Center Nijmegen The Netherlands; ^5^ Donders Centre for Cognitive Neuroimaging, Donders Institute for Brain, Cognition and Behaviour Radboud University Medical Centre Nijmegen The Netherlands; ^6^ Department of Psychiatry Boston Children's Hospital and Harvard Medical School Boston Massachusetts USA; ^7^ Olin Neuropsychiatry Research Center Institute of Living, Hartford Hospital Hartford Connecticut USA; ^8^ Psychiatric Genetics QIMR Berghofer Medical Research Institute Brisbane Queensland Australia; ^9^ Imaging Genetics Center, Mark and Mary Stevens Neuroimaging & Informatics Institute Keck School of Medicine of the University of Southern California Marina del Rey California USA; ^10^ Tri‐institutional Center for Translational Research in Neuroimaging and Data Science (TReNDS) Georgia State University, Georgia Institute of Technology, Emory University Atlanta Georgia USA; ^11^ Department of Psychology and Neuroscience Georgia State University Atlanta Georgia USA; ^12^ Department of Cognitive Neuroscience, Donders Institute for Brain, Cognition and Behaviour Radboud University Medical Centre Nijmegen The Netherlands; ^13^ Karakter Child and Adolescent Psychiatry Nijmegen The Netherlands; ^14^ Clinical Translational Neuroscience Laboratory, Department of Psychiatry and Human Behavior University of California Irvine Irvine California USA; ^15^ Center for the Neurobiology of Learning and Memory University of California Irvine Irvine California USA; ^16^ Department of Human Genetics, Donders Institute for Brain, Cognition and Behaviour Radboud University Medical Center Nijmegen The Netherlands; ^17^ Department of Psychiatry, Donders Institute for Brain, Cognition and Behaviour Radboud University Medical Center Nijmegen The Netherlands; ^18^ Donders Institute for Brain, Cognition and Behavior Radboud University Nijmegen The Netherlands; ^19^ Orygen, The National Centre of Excellence in Youth Mental Health Parkville Victoria Australia; ^20^ Centre for Youth Mental Health The University of Melbourne Melbourne Victoria Australia

**Keywords:** autism spectrum disorder, brain asymmetry, brain laterality, major depressive disorder, mega‐analysis, meta‐analysis, obsessive–compulsive disorder, structural imaging

## Abstract

Left–right asymmetry of the human brain is one of its cardinal features, and also a complex, multivariate trait. Decades of research have suggested that brain asymmetry may be altered in psychiatric disorders. However, findings have been inconsistent and often based on small sample sizes. There are also open questions surrounding which structures are asymmetrical on average in the healthy population, and how variability in brain asymmetry relates to basic biological variables such as age and sex. Over the last 4 years, the ENIGMA‐Laterality Working Group has published six studies of gray matter morphological asymmetry based on total sample sizes from roughly 3,500 to 17,000 individuals, which were between one and two orders of magnitude larger than those published in previous decades. A population‐level mapping of average asymmetry was achieved, including an intriguing fronto‐occipital gradient of cortical thickness asymmetry in healthy brains. ENIGMA's multi‐dataset approach also supported an empirical illustration of reproducibility of hemispheric differences across datasets. Effect sizes were estimated for gray matter asymmetry based on large, international, samples in relation to age, sex, handedness, and brain volume, as well as for three psychiatric disorders: autism spectrum disorder was associated with subtly reduced asymmetry of cortical thickness at regions spread widely over the cortex; pediatric obsessive–compulsive disorder was associated with altered subcortical asymmetry; major depressive disorder was not significantly associated with changes of asymmetry. Ongoing studies are examining brain asymmetry in other disorders. Moreover, a groundwork has been laid for possibly identifying shared genetic contributions to brain asymmetry and disorders.

## Graphical abstract

Left–right asymmetry of the human brain is one of its cardinal features, and also a complex, multivariate trait. Over the last four years, the ENIGMA‐Laterality Working Group has published six studies of grey matter morphological asymmetry in health and disease, based on total sample sizes from roughly 3,500 to 17,000 individuals, which were between one and two orders of magnitude larger than those published in previous decades. Here we review the findings from these six studies.

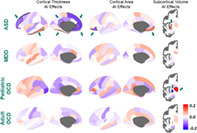

## 1 INTRODUCTION

Left–right asymmetry is an important aspect of human brain organization for multiple functions (Coan & Allen, 2004; Corballis, 2003; Hugdahl & Davidson, 2004; Vigneau et al., 2006; Wheeler, Davidson, & Tomarken, 1993; Zago et al., 2017; Zhen et al., 2017). For example, at least 85% of people have left‐hemisphere language dominance, based on functional magnetic resonance imaging (fMRI) (Mazoyer et al., 2014), and a similar proportion are right‐handed (Gilbert & Wysocki, 1992), although these proportions can vary depending on cut‐off values applied to continuous data (Johnstone, Karlsson, & Carey, 2020). Some anatomical features of the brain are also lateralized at the population level, including the overall “torque” or clockwise twisting of the cerebral hemispheres (viewed from below) (Toga & Thompson, 2003), and the anatomy of cortical regions around the Sylvian fissure (Geschwind & Levitsky, 1968), although again the population proportions depend on cut‐off values, as well as the precise methods for quantifying asymmetry.

The average pattern of human brain asymmetry is established prenatally, as indicated by in utero behavioral data (Hepper, 2013; Parma, Brasselet, Zoia, Bulgheroni, & Castiello, 2017), neuroanatomical studies of fetuses and newborns (Abu‐Rustum, Ziade, & Abu‐Rustum, 2013; Kasprian et al., 2011), and gene expression analyses in which left‐ and right‐sided samples from the embryonic central nervous system were contrasted (de Kovel et al., 2017; Ocklenburg et al., 2017; Sun et al., 2005). However, human brain laterality is also highly variable across individuals, and sizeable proportions of the population can have either more bilateral arrangements, or even reversed asymmetries. For example, roughly 1% of the population has rightward hemispheric language dominance, compared to the majority being leftward lateralized in the population (Mazoyer et al., 2014). Up to 11% of the population have a larger *planum temporale* (a cerebral cortical region located at the posterior end of the sylvian fissure) on the right hemisphere than the left (Geschwind & Levitsky, 1968). It has also become clear in recent years that different aspects of brain asymmetry, such as language dominance and handedness, or structural versus functional measures, can vary largely independently of each other (Mazoyer et al., 2014; Rentería, 2012), such that brain asymmetry must be considered as a complex and multivariate trait.

The extent to which brain asymmetry varies with biological factors such as age, sex, handedness, brain size, and heredity, are open questions (Guadalupe et al., 2017; Kong, et al., 2018; Rentería, 2012). The results of structural magnetic resonance imaging (MRI) studies have often been inconsistent, likely due to small study sample sizes in relation to subtle effects, as well as methodological differences across studies such as differences in scanner hardware, software, and distinct data processing pipelines (Biberacher et al., 2016). Low power in a study not only reduces the chance of detecting true effects, but also the likelihood that statistically significant results reflect true effects (Munafo & Flint, 2010).

Altered hemispheric asymmetry has been associated with numerous brain conditions, including dyslexia (Altarelli et al., 2014), Alzheimer's disease (Thompson et al., 1998), attention‐deficit/hyperactivity disorder (ADHD) (Shaw et al., 2009), psychotic disorders (Crow, 1990; Yucel et al., 2002; Yucel et al., 2003), autism (Eyler, Pierce, & Courchesne, 2012), and mood disorders (Yucel et al., 2009), but the literature has not been consistent (de Kovel et al., 2019; Kong, et al., In press; Postema et al., 2019). In addition to limited sample sizes and methodological heterogeneity, inconsistency across studies has probably arisen due to differences in clinical characteristics, such as comorbidity and medication use. Etiological and neurobiological heterogeneity is also an aspect of these disorders (Carlisi et al., 2017; Jeste & Geschwind, 2014).

In the ENIGMA (Enhancing Neuro‐Imaging Genetics through Meta‐Analysis) consortium (http://enigma.ini.usc.edu), researchers from around the world collaborate to analyze many separate datasets jointly to maximize power of studies, and to reduce some of the technical heterogeneity by using harmonized protocols for MRI data processing (Thompson, et al., 2014; Thompson et al., 2020). In the ENIGMA‐Laterality Working Group, we focus on mapping left–right asymmetry of the brain. This includes measuring the extent to which various factors associate with variability of laterality in the general population and healthy controls, and characterizing differences in laterality in psychiatric disorders. Over the last 4 years, we have carried out studies of brain asymmetry in healthy individuals (Guadalupe et al., 2017; Kong, et al., 2018) and individuals with disorders (de Kovel, Aftanas, et al., 2019; Kong, et al., In press; Postema et al., 2019) using sample sizes roughly 1–2 orders of magnitude larger than previously achieved by the field. We also used summary statistics from the largest of these studies (based on data from over 17,000 participants) to address the critical issue of reproducibility in human neuroscience research (Kong, et al., 2019). In this review, we summarize the general approach taken by our studies of brain asymmetry to date (de Kovel, Aftanas, et al., 2019; Guadalupe et al., 2017; Kong, et al., 2018; Kong, et al., 2019; Kong, et al., In press; Postema et al., 2019), the most important findings and insights gained, as well as potential for future activities by the ENIGMA‐Laterality Working Group.

## 2 T1‐WEIGHTED IMAGE ANALYSIS

All studies by the ENIGMA‐Laterality Working Group thus far were based on structural T1‐weighted brain MRI scans, acquired at multiple study sites around the world, primarily over the last 20 years. The separate datasets were collected through independent studies, without prospective plans for larger‐scale merged or meta‐analyses. Images were acquired using different field strengths (e.g., 1.5 Tesla [T] or 3 T), scanner types, and scanning parameters. However, by participating in ENIGMA studies, each site applied harmonized protocols for data processing and quality control (http://enigma.ini.usc.edu/protocols/imaging-protocols). The protocols were designed to run without the need for sites to send their full‐brain image data to a central analysis group. This approach maximized participants as individual‐level data sharing was restricted due to ethical or consent issues (Thompson, et al., 2014; Thompson et al., 2020).

Cortical parcellations and subcortical segmentations were performed with the freely available and validated software FreeSurfer (versions 5.1 or 5.3) (Fischl, 2012), using the “recon‐all” pipeline, which also incorporates spatial normalization. Thickness and surface area measures for 34 bilaterally paired cortical regions were derived, as defined with the Desikan‐Killiany atlas (Desikan et al., 2006), as well as the average cortical thickness and total surface area per hemisphere. In addition, left and right volumes of seven bilaterally paired subcortical structures were obtained (or sometimes eight structures including the lateral ventricles, if those data were available). Parcellations of cortical gray matter regions, and segmentations of subcortical structures, were visually inspected following the standardized ENIGMA quality control protocol (http://enigma.ini.usc.edu/protocols/imaging-protocols). Briefly, cortical segmentations were overlaid on the T1‐weighted image of each subject. Web pages were generated with snapshots from internal slices, as well as external views of the segmentation from different angles. All sites were provided with a manual on how to assess the quality of these images, including examples of common segmentation errors. For subcortical structures, the protocol again consisted of visually checking the individual images, plotted from a set of internal slices. Volume estimates derived from poorly segmented structures (i.e., where tissue labels were assigned incorrectly) were excluded from each site's datasets and subsequent analyses. In addition, any data points exceeding 1.5 times the interquartile range, as defined per site and diagnostic group, were visually inspected (in 3D). When identified as error, all values from the affected regions were excluded from further analysis.

## 3 ASYMMETRY INDEXES

Subject‐specific asymmetry indexes, *AI* = (Left–Right)/(Left+Right), were derived for each brain regional and global hemispheric measure. The *AI* is a widely used measure in brain asymmetry studies (Kurth, Gaser, & Luders, 2015; Leroy et al., 2015). The denominator ensures that the index does not simply scale with brain size. Note that other similar definitions of the *AI* can sometimes be used, for example with an additional scaling factor of 2, that is, (Left–Right)/((Left+Right)/2), or else using Right–Left as the numerator instead of Left–Right. However, these variants of the *AI* all deliver essentially the same findings. We considered a region to show population‐level laterality whenever the mean AI was significantly different from zero (or in some studies when a paired *t* test to compare left and right measures showed a significant difference).

The Desikan‐Killiany atlas (Desikan et al., 2006) was derived from manual segmentations of sets of reference brain images. The labeling system incorporates hemisphere‐specific information on sulcal and gyral geometry with spatial information regarding the locations of brain structures, and shows a high accuracy when compared to manual labeling results (Desikan et al., 2006). Accordingly, the mean regional asymmetries in our datasets might partly reflect left–right differences present in the reference dataset used to construct the atlas. For detecting cerebral asymmetries with automated methods, some groups have chosen to work from artificially created, left–right symmetrical atlases (Kawasaki et al., 2008). However, our studies were focused primarily on comparing *relative* asymmetry between groups, or in relation to continuous predictors. The use of a “real‐world” asymmetrical atlas had the advantage that regional identification is likely to be accurate for structures that are asymmetrical both in the atlas and, on average, in our datasets.

It is possible that the quality control procedure outlined in the previous section may have slightly affected the population‐level average asymmetry estimates, if one hemispheric measure was particularly affected by poor segmentation performance and resulting data exclusion in a minority of participants. However, we only computed asymmetry indexes per subject when measurements were present from both hemispheres. The QC procedure was designed to be practical for processing thousands of participants, each with multiple parcellated regions. Manual correction in case of visible software errors was not feasible on this scale, and manual segmentation is not free from bias either (Maltbie et al., 2012).

## 4 TESTING FOR FACTORS THAT AFFECT BRAIN ASYMMETRY

For studies in which Freesurfer‐derived data were available from all sites to be shared with a central analysis group (de Kovel, Aftanas, et al., 2019; Kong, et al., In press; Postema et al., 2019), linear mixed‐effects random intercept models were fitted separately for each cortical regional surface and thickness *AI*, as well as the total hemispheric surface area and mean thickness *AI*, and the subcortical volume *AI*s. This was performed using a function such as “nlme” in R (Pinheiro, et al., 2018). A typical base model was:

$$\mathrm{AI}=\text{trait}+\mathrm{sex}+\mathrm{age}+\text{dataset}\left(\text{random}\right)$$

In these models, “AI” was the asymmetry index of a given brain structure. “Trait” was the trait of interest being tested, such as a binary fixed effect for case–control status in a disorder study. “Sex” was a binary fixed effect, “age” was a numeric fixed effect, and “dataset” was a random effect with as many categories as there were separate datasets in the study. Age was significantly associated with some asymmetry measures in our study of >17,000 participants from the general population and healthy controls (see below). We therefore included age as a covariate in all of our subsequent studies, such as those of disorders. Significance was assessed based on the *p*‐value for the effect of the trait of interest on a given *AI*. Multiple testing correction over multiple AIs was performed either by Bonferroni correction, or using the False Discovery Rate (FDR) (Benjamini & Hochberg, 1995). The two approaches were similar for these data because different structural AIs tended to be only weakly correlated with each other.

Secondary analyses, using more complex models, were applied as appropriate to the particular study questions. For example, psychiatric disorders can involve sex‐ or age‐differences in prevalence or presentation, and because of this, models that included sex or age interaction terms were fitted. Nonlinear age effects were also fitted, although these were found generally to be of little relevance to brain asymmetries (de Kovel, Aftanas, et al., 2019; Kong, et al., In press; Postema et al., 2019). For studies of disorders, there were various clinical variables present, such as medication use, acute versus remission status, first episode versus recurrent episodes, age at onset, and disorder severity (de Kovel, Aftanas, et al., 2019; Kong, et al., In press; Postema et al., 2019).

Sensitivity analyses were performed according to the issues relevant to each separate study. For example, in the study of ASD we repeated the main analyses after having removed very young participants, as segmentation might have been especially challenging for the FreeSurfer algorithms (Postema et al., 2019). We also repeated the main analyses after removing the subset of data acquired at 1.5 T (the majority of datasets were collected at 3 T), to test for possible sensitivity to this technical variable (Postema et al., 2019).

Not all sites were able to share derived Freesurfer variables for analysis by a central group. Therefore, to increase participation for some of our studies (Guadalupe et al., 2017; Kong, et al., 2018), we instead took an approach based on meta‐analytic techniques. For these studies, the separate sites ran linear modeling on their own data, and then shared summary statistics with the central group for meta‐analysis. For example, in our study of cerebral cortical asymmetries in 99 datasets comprising population data or healthy controls (Kong, et al., 2018), we combined summary statistics from each dataset using inverse variance‐weighted random‐effect meta‐analyses (Borenstein, Hedges, Higgins, & Rothstein, 2010). This method tests one overall effect, while weighting each dataset's contribution by the inverse of its corresponding sampling variance. Test statistics in the meta‐analyses were computed based on a standard normal distribution. As including results based on too few participants may reduce reliability, we only included datasets with a sample size larger than 15. In the meta‐analysis, heterogeneity of each effect was assessed via the *I* ^2^ value (Higgins, Thompson, Deeks, & Altman, 2003), which describes the percentage of total variation across studies that is due to heterogeneity, rather than chance.

Although a single image analysis pipeline was applied to all datasets, heterogeneity of imaging protocols was a feature of these studies. There were substantial differences between datasets in the average asymmetry measured for some regions, which may be due in part to different scanner characteristics, as well as differences in demographic or disorder patient profiles. Properties of MRI scanners such as field strength, manufacturer, gradient nonlinearity, subject positioning, and longitudinal drift have been long understood to increase bias and variance in the measurement of brain structural measures (Fortin et al., 2018). We either corrected statistically for “dataset” as a random effect in our models, or else this was accounted for implicitly in the meta‐analytic studies. However, it is possible that between‐dataset variability results in reduced statistical power, relative to hypothetical, equally‐sized, single‐center studies. In reality, few single centers have been able to collect such large samples alone. As long as researchers publish many separate papers based on single datasets, collected in particular ways, the field overall has the same problem. In this case, multicenter studies can better represent the real‐world heterogeneity, typically with more generalizable findings than single‐center studies (Costafreda, 2009). The primary purpose of our studies, based on multiple datasets originally collected as separate studies, was to assess the total combined evidence for effects over all available datasets, while allowing for heterogeneity between datasets, and including sensitivity and secondary analyses with respect to relevant variables.

## 5 FINDINGS IN GENERAL POPULATION AND HEALTHY CONTROL DATA

### 5.1 Cerebral cortical asymmetries

We carried out the largest ever analysis of cerebral cortical asymmetry and its variability across individuals (Kong, et al., 2018), based on 17,141 individuals from 99 datasets worldwide, from diverse ethnic backgrounds. Participants were drawn from the general population, or were healthy controls from clinical studies. Prior findings in the literature were based on sample sizes no greater than the low hundreds, and using different methods (Kong, et al., 2018). Our large‐scale study improved on this situation and achieved a more accurate description of the average asymmetries of the healthy human brain, as well as variation in these asymmetries, and some factors that affect individual differences in them. Image processing and effect size estimations were conducted at each participating site, and output statistics from each dataset were combined using random‐effect meta‐analysis (see above). All of the meta‐analytic effect sizes and confidence intervals from that study can be found at this website: conxz.net/neurohemi.

**FIGURE 1:**
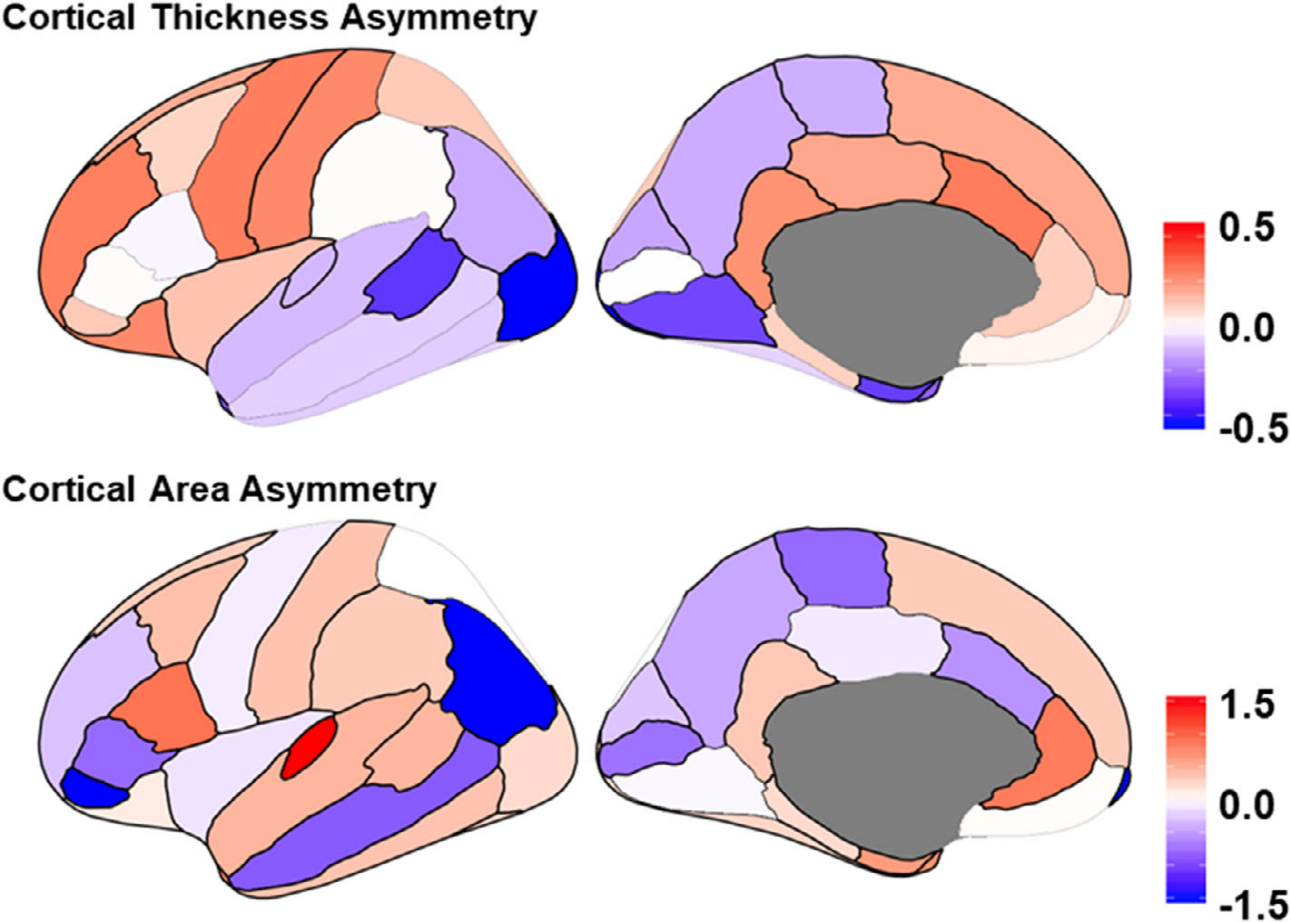
Population average regional asymmetries of cortical thickness, and surface area. Colors indicate the directions and effect sizes (Cohen's *d*) of average interhemispheric differences, with red indicating leftward asymmetry (i.e., a greater left‐side than right‐side measure), and blue indicating rightward asymmetry. The maps were created using the “ggseg” R package based on meta‐analyzed data from more than 17,000 subjects (Kong, et al., 2018)

At the whole‐hemisphere level, it was revealed that, on average, the left hemisphere has a generally thicker cortex but smaller surface area than the right (Figure 1). Regions with significant leftward asymmetry in thickness (i.e., left > right) were identified mainly in the frontal cortex, as well as the primary sensory, superior parietal, and medial temporal cortices. Rightward thickness asymmetry was prominent in the posterior cortex, including lateral and medial regions of the temporal, parietal, and occipital cortices. Considered all together, there was a striking asymmetry pattern along the fronto‐occipital axis (Figure 1), which may be related to “Yakovlevian torque”, that is, the frontal/occipital bending in the human brain (Yakovlev, 1972).

Regarding surface area, population‐level average asymmetry was generally more prominent compared to that of cortical thickness. A large majority of regions showed significant asymmetry in surface area, although with no obvious directional pattern affecting neighboring regions, or along the anterior–posterior axis, as we observed for thickness. We identified several regions that are asymmetric in surface area that had not previously been described as such. Among these regions, two language‐related regions, that is, the opercular part of the inferior frontal gyrus (the posterior part of Broca's area) and the transverse temporal gyrus (Heschl's gyrus) showed the largest leftward asymmetries of surface area. These population‐level, average asymmetries of surface area may contribute to the typical leftward lateralization of language in these regions. However, we found two other language‐related regions showing strong asymmetry of surface area in the opposite direction (i.e., right > left), which were the *pars triangular is* of the inferior frontal gyrus (the anterior part of Broca's area) and the inferior parietal gyrus. Therefore, any macro‐anatomical basis of functional language lateralization must be more complex than a straightforward, relatively enlargement of left‐hemisphere classical language regions. We did not find a significant average cortical thickness asymmetry in the *pars opercularis* or *pars triangularis* of the inferior frontal gyrus, in contrast to a study by Plessen et al. in 215 healthy participants (Plessen, Hugdahl, Bansal, Hao, & Peterson, 2014), that had suggested thickness asymmetry of these regions to be an anatomical reflection of left‐hemisphere language dominance.

There was no clear association of cerebral cortical asymmetry measures with handedness (Kong, et al., 2018) (555–608 left‐handers vs. 6,222 to 7,243 right‐handers, depending on the specific regional asymmetry measure), which underlines that structural and functional lateralities can vary largely or wholly independently (see Section 7). However, various regional cortical surface area and thickness asymmetries were related to sex, age, or intracranial volume (Kong, et al., 2018). Notably, we found no average sex differences in cortical thickness asymmetry of core regions of the language network, including the *pars opercularis* and *pars triangularis*, transverse temporal gyrus, and supramarginal gyrus. This indicates that subtle sex differences in performance on language tasks, and in language lateralization (Clements et al., 2006), are not linked to sex differences in cortical thickness asymmetry of these regions, in contrast to a prior suggestion by Plessen et al. (2014).

Age was positively correlated with more pronounced leftward asymmetry of total hemispheric cortical thickness, an effect to which the superior temporal gyrus made a particularly large contribution. Again, regional effects of age on asymmetry did not match well with results previously found in 215 subjects by Plessen et al. (Plessen et al., 2014).

We also found that leftward asymmetry in cortical thickness is greater in larger brains, an effect that was the most pronounced in the inferior parietal gyrus and the insula. One possible explanation is that increased interhemispheric distance and transfer time in larger brains favors increased hemispheric differentiation, and therefore greater asymmetries (Herve, Zago, Petit, Mazoyer, & Tzourio‐Mazoyer, 2013).

As part of this study, we also analyzed two independent twin and/or family datasets, to estimate maximal heritabilities of cortical asymmetry measures. Several regional asymmetries (e.g., parahippocampal thickness asymmetry and superior temporal area asymmetry) showed significant and replicable heritability across these two datasets. These results provide a basis for future studies on the molecular genetic contributions to brain asymmetry, and possible genetic correlations with cognitive, neurological, and psychiatric disorders.

### 5.2 Reproducibility of cortical asymmetry across datasets

The issue of reproducibility has received considerable attention in a variety of fields including medicine (Prinz, Schlange, & Asadullah, 2011), psychology (Aarts, et al., 2015; Klein et al., 2014), and neuroscience (Button et al., 2013). Poor reproducibility has been partly attributed to the file‐drawer problem, through which unwanted results can sometimes remain unpublished, as well as problematic practices such as selecting only those statistical analyses for inclusion in publications that yield positive results (*p*‐hacking) (Aarts, et al., 2015; Baker, 2016; Bakker, van Dijk, & Wicherts, 2012; Ioannidis, 2005; Ioannidis, 2008; Ioannidis, Munafo, Fusar‐Poli, Nosek, & David, 2014; John, Loewenstein, & Prelec, 2012; Simmons, Nelson, & Simonsohn, 2011). Low statistical power in individual studies is also an important factor (Button et al., 2013; Ioannidis, 2005). We carried out an empirical illustration of reproducibility in the absence of publication bias or *p*‐hacking, by re‐analyzing the summary statistics from our study of cerebral cortical asymmetry in 99 datasets (Kong, et al., 2018; Kong, et al., 2019). For this purpose, we considered the meta‐analytic hemispheric effect sizes (i.e., population‐level asymmetry measures) to be “true.” The results within each separate dataset were then viewed as coming from separate studies in an “ideal publishing environment,” that is, free from selective reporting and *p*‐hacking. This was because the study was not a literature‐based meta‐analysis, but made use of 99 datasets that were contributed specifically for this study, without prior measurement of asymmetry. A hemispheric effect was considered to be reproduced in a given dataset when it was found with unadjusted *p* < .05 and in the same left–right direction as the meta‐analysis effect in all the other 98 datasets. This would be a typical threshold used, if each dataset had been studied separately, and its findings published separately.

We found that the average reproducibility rate, over all regional and total hemispheric effects, was limited (mean = 65.28%, *SD* = 23.86%, min = 23.2%, max = 100%). As expected, reproducibility increased with the “true” (i.e., meta‐analytic) effect size, as well as the sample sizes of the datasets, which together contribute to statistical power. These findings constitute an informative illustration, as they reflect realistic biological effects in heterogeneous neuroscience data, and in typically‐used sample sizes. In this way, the ENIGMA‐Laterality Working Group has helped to increase awareness of these importantly and timely issues in the broader field of neuroscience.

### 5.3 Subcortical volume asymmetries

**FIGURE 2:**
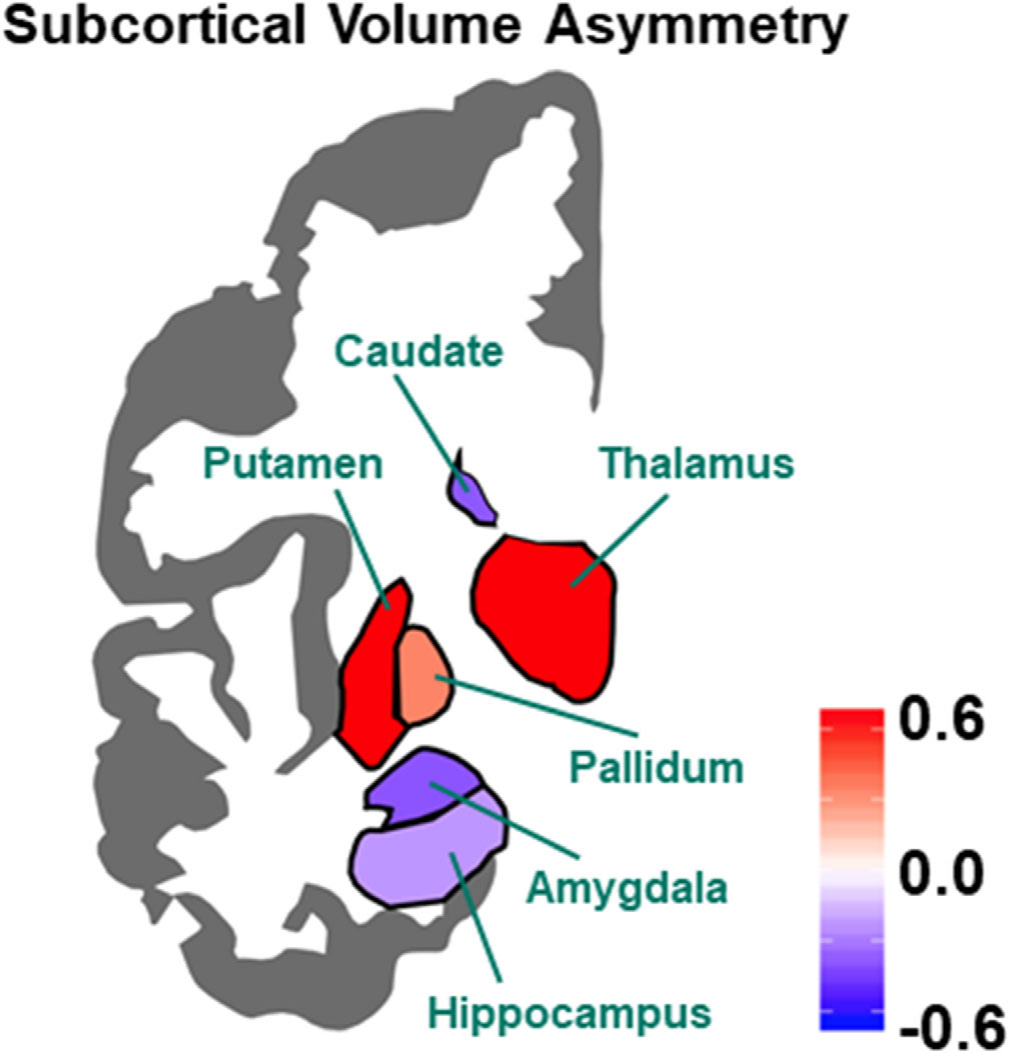
Population average regional asymmetries of subcortical volumes. Colors indicate the directions and effect sizes (Cohen's *d*) of average inter‐hemispheric differences; red indicates leftward asymmetry (i.e., a greater left‐side than right‐side measure), and blue indicates rightward asymmetry. The maps were created using the “ggseg” R package based on meta‐analyzed data from more than 15,000 subjects (Guadalupe et al., 2017)

Lateralities of human subcortical and hippocampal volumes, and the factors that might affect their individual differences or roles in lateralized cognition, are less well studied than of the cerebral cortex. The literature prior to 2017, based on limited sample sizes, was extremely inconsistent with regard to possible effects of sex, age, and handedness (Guadalupe et al., 2017). We carried out a study that was, by two orders of magnitude, the largest of subcortical asymmetries (Figure 2) (Guadalupe et al., 2017). This was again a harmonized multi‐site study using meta‐analysis methods (Guadalupe et al., 2017). Volumetric asymmetries of seven subcortical structures were assessed in 15,847 MRI scans, from 52 datasets worldwide.

At the population level, all subcortical structures showed significant asymmetries of volume (Figure 2), with the thalamus, putamen, and pallidum having larger average volumes in the left hemisphere, and the hippocampus, amygdala, nucleus accumbens, and caudate nucleus having larger volumes in the right hemisphere (Figure 2). Handedness was not associated with subcortical asymmetries, even in this unprecedented sample size. There were sex differences in the asymmetry of the globus pallidus and putamen. For the putamen, this involved a rightward shift in asymmetry in males relative to females. The opposite was found for the globus pallidus, where a leftward shift in asymmetry was observed in males. For the putamen, there was also a leftward shift in asymmetry with increasing age. Meanwhile, various previously claimed effects of age and sex on subcortical asymmetries were not supported (Guadalupe et al., 2017), which likely indicates the problematic nature of a literature based on small sample sizes.

As part of this study, we also measured the maximal heritabilities of subcortical and hippocampal asymmetries in a large dataset of extended families (McKay et al., 2014). Asymmetries of the globus pallidus, hippocampus, putamen, and thalamus showed significant heritabilities ranging from 0.15 to 0.27. As in our cortical study (above), the heritability analysis can be a basis for future genome‐wide association studies, with eventual potential to test for genetic overlap between these asymmetries and cognitive or psychiatric disorders.

## 6 FINDINGS FROM DISORDER CASE–CONTROL STUDIES

### 6.1 Autism spectrum disorder

Functional imaging data have indicated that people with ASD have reduced leftward language lateralization more frequently than healthy controls (Kleinhans, Muller, Cohen, & Courchesne, 2008; Knaus et al., 2010; Lindell & Hudry, 2013). Resting‐state functional MRI of people with ASD has also suggested a rightward shift of asymmetry that involves various functional networks (Cardinale, Shih, Fishman, Ford, & Muller, 2013). People with ASD have a higher rate of left‐handedness than the general population (Lindell & Hudry, 2013; Markou, Ahtam, & Papadatou‐Pastou, 2017; Rysstad & Pedersen, 2018). In addition, brain structural imaging studies have reported altered hemispheric asymmetry in ASD, including studies of white matter tracts (Carper, Treiber, DeJesus, & Muller, 2016; Conti et al., 2016; Joseph et al., 2014), gray matter volume, surface and thickness (Dougherty, Evans, Katuwal, & Michael, 2016; Floris et al., 2016).

However, prior to 2019, studies of brain structural asymmetry in ASD had sample sizes of less than 128 cases and 127 controls, and results were inconsistent (Postema et al., 2019). We made use of MRI data from 54 datasets that were collected across the world by members of the ENIGMA consortium's ASD Working Group, to perform the first highly powered study of structural brain asymmetry in ASD. Derived data via Freesurfer were made available from 1,774 individuals with ASD and 1,809 controls, from the 54 datasets combined. Therefore, it was possible to analyze these data using a mega‐analytic approach, applying linear mixed‐effect models, including a random intercept variable for “dataset” (see above). The ASD participants had predominantly DSM‐IV diagnoses of “Autistic Disorder,” rather than milder spectrum disorders.

**FIGURE 3:**
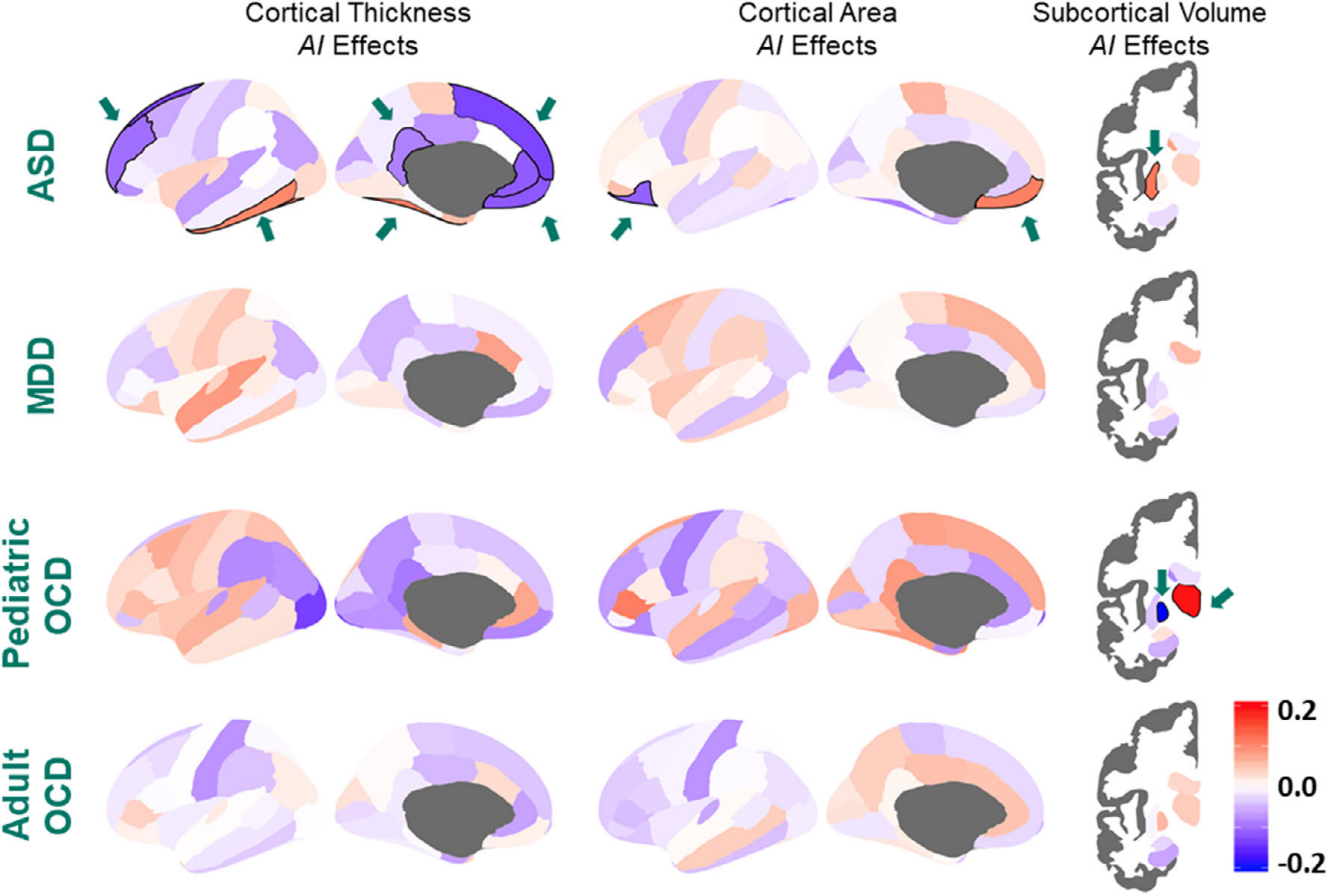
Brain asymmetry in disorders, as compared to healthy controls. Cohen's *d* effect sizes of the associations between disorder diagnosis and *AI*s. The *d* values are overlaid on the left hemisphere for visualization. Positive Cohen's *d* values (red) indicate mean shifts towards greater leftward or reduced rightward asymmetry in cases relative to controls, and negative Cohen's *d* values (blue) indicate mean shifts towards greater rightward asymmetry or reduced leftward asymmetry in cases relative to controls. Significant effects after FDR correction in each study are highlighted by a black boundary and a green arrow (i.e., cortical asymmetry effects for ASD and subcortical asymmetry effects for ASD and pediatric OCD). The maps are reproduced from data in de Kovel, Aftanas, et al. (2019), Kong et al. (In press), and Postema et al. (2019). Sample sizes were up to: 1773 ASD versus 1,722 controls, 2,254 MDD versus 3,504 controls (cortical), 2,540 MDD versus 4,230 controls (subcortical), 501 pediatric OCD versus 439 controls, 1,777 adult OCD versus 1,654 controls

ASD was significantly associated with alterations of cortical thickness asymmetry in mostly medial frontal, orbitofrontal, cingulate, and inferior temporal regions, as well as with asymmetry of orbitofrontal surface area (Figure 3). The case–control average differences generally involved lower asymmetry in individuals with ASD compared to controls. In addition, putamen volume asymmetry was altered in ASD. However, the largest case–control effect size was Cohen's *d* = −0.13, for asymmetry of the superior frontal cortical thickness. This finding indicates that large‐scale analysis was necessary to quantify very small alterations of average brain structural asymmetry in ASD. Most effects did not depend on age, sex, IQ, ASD severity, or medication use.

Given the very small effect sizes, structural brain asymmetry alone is unlikely to be a useful biomarker for ASD, in terms of individual‐level prediction or diagnosis. Prior studies using smaller samples were clearly underpowered in this context, and their relatively large claimed effects are likely to have been false positives. Alternatively, prior effects reported in the literature may be restricted to particular patient subgroups, or else not discernible with the imaging analysis pipeline used in our study.

Regardless of small effect sizes, our findings inform understanding of the neurobiological underpinnings of ASD. As the bulk of the datasets comprised children (Postema et al., 2019), the findings suggest that altered lateralized neurodevelopment may be a feature of ASD, affecting widespread brain regions with diverse functions. Some of the affected cortical regions are involved in social cognitive processes (Adolphs, 2009), including perceptual processing (fusiform gyri), cognitive and emotional control (anterior cingulate) and reward evaluation (orbitofrontal cortex, ventral striatum). However, the roles of these brain structures are not restricted to social behavior, and various additional regions were also affected. Many of the affected regions, including medial frontal, anterior cingulate and inferior temporal regions, overlap with the default mode network (DMN) (Raichle, 2015). DMN organization has shown evidence for differences in ASD (Carlisi et al., 2017; Christakou, et al., 2013; Nunes, Peatfield, Vakorin, & Doesburg, 2019; Uddin, 2011), including alterations in functional laterality (Nielsen et al., 2014). Our findings may therefore support a role of altered lateralization of the DMN in ASD.

### 6.2 Major depressive disorder

Studies using dichotic listening, visual hemifield analysis, electro‐encephalography, and neuroimaging, have reported changes of neurophysiological asymmetry between individuals with MDD and healthy controls, particularly involving reductions of left frontal and right parieto‐temporal function in depressive disorders (Bruder, Stewart, & McGrath, 2017; Coan & Allen, 2003; Davidson, 1998; Jesulola, Sharpley, Bitsika, Agnew, & Wilson, 2015; van der Vinne, Vollebregt, van Putten, & Arns, 2017). Such neurophysiological changes might conceivably be reflected in terms of altered structural asymmetry; for example, the number of pyramidal cells is thought to influence cortical EEG recordings (Kenemans, 2013), while a difference in the number of pyramidal cells may also affect cortical thickness (Shin et al., 2004). In fact, an inverse relation between cortical thickness and EEG alpha power has been reported for some cortical regions (Bruder et al., 2012). However, prior to 2019, brain structural asymmetry in MDD had only been investigated in a small number of individual studies, with total sample sizes less than 100.

We investigated structural asymmetry in up to 2,540 MDD individuals and 4,230 controls, from 32 datasets included in the ENIGMA‐MDD Working Group (de Kovel, Aftanas, et al., 2019). Derived Freesurfer data were made available to a central analysis group for linear mixed‐effect modeling, again including a random intercept variable for “dataset” (see above). The unprecedented sample size provided 80% power to detect effects of the order of Cohen's *d* = 0.1.

However, the largest effect size of MDD diagnosis was Cohen's *d* = 0.085 for the thickness asymmetry of the superior temporal cortex, which was not significant when adjusting for multiple testing (Figure 3). We found no support for alterations of asymmetry that are consistent with those reported in two previous, small studies of the dorsolateral prefrontal cortex (Liu et al., 2016) or frontal lobe (Kumar, Bilker, Lavretsky, & Gottlieb, 2000). Asymmetry measures were also not significantly associated with medication use, acute versus remitted status, first episode versus recurrent status, or age at onset.

The possibility remains that brain functional or structural asymmetry might be altered in MDD in some etiological subgroups. However, a recent meta‐analysis of frontal alpha asymmetry as a diagnostic marker in depression (16 studies, MDD: *n* = 1,883, controls: *n* = 2,161) found no significant difference between groups of individuals with MDD and controls (van der Vinne et al., 2017). Altered brain anatomical and neurophysiological asymmetry may therefore be of little relevance to MDD etiology in most cases. Our study illustrates the importance of taking large‐scale and systematic approaches to the study of brain‐disorder associations.

### 6.3 Obsessive compulsive disorder

Altered functional laterality has been investigated in OCD (Abramovitch, Abramowitz, & Mittelman, 2013; Kuelz, Hohagen, & Voderholzer, 2004), partly stemming from observations of psychometric deficits within the visual–spatial domain (typically rightward lateralized in healthy people) (Kuskowski et al., 1993; Maril, Hermesh, Gross‐Isseroff, & Tomer, 2007; Rao et al., 2015), as well as altered emotional processing (again some aspects of emotion are typically rightward lateralized) (Ischebeck, Endrass, Simon, & Kathmann, 2014; Schienle, Schafer, Stark, Walter, & Vaitl, 2005; Simon, Kaufmann, Musch, Kischkel, & Kathmann, 2010; Wexler & Goodman, 1991). However, left‐sided dysfunction has also been suggested in OCD, on the basis of neuropsychological data (Wexler & Goodman, 1991) as well as electrophysiological data (Shagass, Roemer, Straumanis, & Josiassen, 1984; Shin, Ha, Kim, & Kwon, 2004; Tot, Ozge, Comelekoglu, Yazici, & Bal, 2002).

Prior to 2019, two previous studies had explored brain structural asymmetry in OCD as a specific outcome of interest, but the larger of these had only 32 affected people (Garber, Ananth, Chiu, Griswold, & Oldendorf, 1989; Peng et al., 2015). To remedy this, we conducted a study of structural brain asymmetry using 16 pediatric datasets (<18 years old; 501 OCD patients and 439 healthy controls), as well as 30 adult datasets (≥18 years old; 1,777 patients and 1,654 controls) (Kong, et al., In press). Data were analyzed separately in these two age groups because the ENIGMA‐OCD Working Group had previously indicated distinct alterations in pediatric and adult patients (Boedhoe, et al., 2017; Boedhoe, et al., 2018).

Linear mixed‐effect modeling was used to test for case–control differences, including a random intercept variable for “dataset” (see further above).

In the pediatric datasets, the largest case–control differences were observed for volume asymmetry of the thalamus (more leftward; Cohen's *d* = 0.19) and the pallidum (less leftward; *d* = −0.21) (Figure 3) (Kong, et al., In press). No significant case–control differences were found in the adult datasets. The thalamus is involved in diverse interactions among cortical, subcortical, and brainstem nuclei, and many of its functions are asymmetrical in healthy subjects (Ojemann, 1977). A subtle change of thalamus asymmetry in pediatric patients is broadly in accordance with previous disease models for OCD as regards the cortico‐striato‐thalamo‐cortical (CSTC) circuitry, which is involved in a wide range of cognitive, motivational and emotional processes (van den Heuvel et al., 2016). However, it is not clear what specific pathophysiologic mechanisms might link altered thalamus asymmetry to OCD. Within OCD individuals, we found no associations of thalamus asymmetry with medication status, age at a disease onset, disease duration, current anxiety and depression comorbidity, or disease symptom dimensions. As the thalamus is subdivided into cytoarchitectonically distinct nuclei with different functions (Behrens et al., 2003), future studies using higher resolution mapping of internal thalamus structure and function might be informative in pediatric OCD.

As regards the pallidum, this structure links with the striatum and thalamus within the CSTC circuitry (van den Heuvel et al., 2016), and has roles in reward and motivation, as well as broader cognitive, affective, and sensorimotor processes (Smith, Tindell, Aldridge, & Berridge, 2009; van den Heuvel et al., 2016). While it is not clear why lateralized changes in particular should be involved in OCD, our findings in pediatric cases help to characterize the brain structural changes in this disorder, and suggest altered laterality of subcortical neurodevelopment affecting CSTC circuitry.

### 6.4 Disorder studies in progress

We currently have two case–control disorder studies of brain asymmetry underway, one for ADHD, the other for schizophrenia. The ADHD study is based on up to 1,978 cases and 1,917 controls, from 39 datasets, and linear mixed effect modeling is being used, with a random intercept variable for “dataset.” In contrast, the study of schizophrenia is based on meta‐analysis methodology (see above), whereby each separate contributing group sends summary statistics to a central group, rather than their derived Freesurfer data. The total sample size is not yet known.

## 7 CONCLUSIONS AND PROSPECTS

The ENIGMA‐Laterality Working Group has mapped gray matter morphological asymmetry in health and disease on a larger scale than ever before. An improved description of the healthy brain's typical asymmetrical form has been achieved, together with realistic estimates of the extent to which different biological factors and disorders are associated with variance in brain asymmetry. The studies have illustrated how high‐powered and systematic studies can yield much needed clarity in human neuroscience, where prior smaller and more methodologically diverse studies produced inconsistent results.

In our studies we have generally not attempted detailed reviews of previous findings in the literature, contrasted with our findings (although we mentioned specific examples in the sections above). In relation to disorders, for example, our findings indicate very small effect sizes for associations with altered brain asymmetry. As the effect sizes in our studies were estimated based on large sample sizes, relatively accurate estimations of the true effects were possible, whether they were statistically significant or not. It is clearly unlikely that such effects would have been detected in previous studies that were one or two orders of magnitude smaller, in terms of sample size. To the extent that true effects are generally of the magnitudes that we have reported, this also means that many effects reported in smaller studies are unlikely to be true. As noted in the Introduction, low power can cause failure to detect true effects, but also reduces the likelihood that significant results reflect true effects (Button et al., 2013; Munafo & Flint, 2010). Our study of the reproducibility of cortical hemispheric differences was informative in this regard, which clearly illustrated the link between sample size and reproducibility (Kong, et al., 2019).

However, in addition to limited sample sizes, there are various other possible explanations for discrepancies with previous studies. Methodological differences in hardware, software, and data processing pipelines can influence results (Biberacher et al., 2016). In terms of brain structural quantification, the cortical atlas that we used did not have perfect equivalents for some regions or measures defined in many of the earlier studies. For example, we did not consider gyral/sulcal patterns or cortical gray matter volumes as such. Rather, we studied regional cortical thicknesses and surface areas as distinct measures, which together drive gray matter volumetric measures. Since area and thickness have been shown to vary relatively independently (Grasby, et al., 2020; Kong, et al., 2018; Panizzon et al., 2009), separate analyses are advisable, although this comes at a cost to statistical power due to increased multiple testing. Investigation with more fine definitions of regions (e.g., sub‐regions of the thalamus [Johansen‐Berg et al., 2005]), or an atlas‐free, vertex‐wise approach to the cerebral cortex combined with cross‐hemispheric registration methods, will likely be useful for future studies of asymmetry (Maingault, Tzourio‐Mazoyer, Mazoyer, & Crivello, 2016; Van Essen, Glasser, Dierker, Harwell, & Coalson, 2012).

The conceptualization of laterality can also differ across studies. For example, some studies have also calculated the unsigned magnitudes of AIs (i.e., absolute degree of asymmetry, regardless of direction) (Douglas et al., 2018). In our studies, we did not calculate absolute AIs, partly not to increase multiple testing, but also because these measures are often highly non‐normal with a floor effect at value zero, which would violate the assumptions of the modeling that we applied. Future studies may consider the unsigned magnitude of brain asymmetry indexes further in ADHD, but it will be necessary to use statistical methods that can account for non‐normal distributions.

Discrepancies with earlier studies may also be due to differences in clinical features of disorders that arise from case recruitment and diagnosis, for example with respect to medication use (Nakao, Radua, Rubia, & Mataix‐Cols, 2011; Pretus et al., 2017), comorbidities (Reale et al., 2017), symptom severity, and/or IQ. For example, it remains possible that certain subsets of ASD might be associated more strongly with altered brain asymmetry than was apparent in our large‐scale analysis of average changes over many datasets, comprising many and varied collections of ASD individuals and controls. In general, between‐center heterogeneity (in terms of methods used, patient subgroups, demographics) may result in reduced statistical power to detect effects that are specific to certain subgroups of datasets, or to individual datasets, when tested in analysis over all datasets. In our studies of disorders, we used random‐intercept models to adjust for heterogeneity between datasets, but this cannot fully rescue power in the case that effects are truly specific to certain subsets. However, no single center has been able to collect such a large disorder case–control datasets alone, while our large sample sizes yielded more precise estimates of effect sizes with respect to overall case–control populations, as represented across many research centers. In this way, findings from multicenter studies such as ours can be considered more generalizable than single‐center studies (Costafreda, 2009). In any case, as long as researchers publish separate papers based on many single, smaller datasets, collected in particular ways, the field overall has the same issue of heterogeneity.

While it is clear that the small effects we found in the disorder case–control studies will not provide useful biomarkers or clinical predictors of disorder, they may nonetheless give insights into aspects of disorder neurobiology. For example, altered average cortical thickness asymmetry in ASD, even though cases and controls largely overlap in terms of variation, may indicate a subtle disruption of lateralized brain development that manifests more relevantly for the disorder at deeper structural or functional levels. The ENIGMA‐Laterality studies so far have been based on morphometric measures of gray matter. Other aspects of structural asymmetry, such as white matter tracts, remain to investigate on this scale, as do higher‐resolution aspects of gray matter structure such as laminar organization and cytoarchitecture. Functional asymmetries should also be investigated on a large scale, but this is more difficult to achieve than for structural data, because there are fewer datasets available within a given task‐functional domain, such as language or attention. In contrast, structural T1 scans tend to be collected in both structural and functional studies, and regardless of the particular functional domain under study. Resting‐state fMRI may be one way to move forward with functional analyses on a large scale, as the intrinsic connectivity networks derived from rs‐fMRI are fairly robust to technical differences between studies (Zuo & Xing, 2014), and also related to task‐functional networks (Cole, Bassett, Power, Braver, & Petersen, 2014).

In general, relations between structural and functional variability of the brain are subtle and complex (Batista‐Garcia‐Ramo & Fernandez‐Verdecia, 2018; Chen & Omiya, 2014; Tzourio‐Mazoyer, Crivello, & Mazoyer, 2018). The population average asymmetrical pattern of human brain structure and function is likely to arise due to a genetically regulated program (see Section 1), which gives probabilistic biases to various aspects of differential development of the two hemispheres. We consider that asymmetrical structure is likely to be linked to function in the typical, population‐average form: for example, the average perisylvian asymmetries of cortical regional thickness and surface area may partly reflect functional laterality for language (see Section 5). However, when the complex, multi‐component program of asymmetrical development is perturbed (perhaps most often by chance in the early embryo) (de Kovel, Carrion‐Castillo, & Francks, 2019; de Kovel & Francks, 2019; McManus, Davison, & Armour, 2013; Postema, Carrion‐Castillo, Fisher, Vingerhoets, & Francks, 2020), then different aspects of asymmetrical organization can apparently vary largely independently in the population, given that low correlations tend to be found between different pairs of asymmetry measures (structure–structure, structure–function or function–function). The low correlations between variance in different aspects of asymmetry may reflect the brain's capacity for plasticity to retain healthy function, and do not necessarily mean that asymmetrical structure and function are unrelated in the population's average form.

To help clarify these issues, the field needs to work towards identifying the genetic‐developmental mechanisms of human brain asymmetry, which are currently poorly understood. Some of the brain asymmetry measures examined here have heritabilities up to roughly 25% (Guadalupe et al., 2017; Kong, et al., 2018), so that gene mapping approaches for different aspects of brain asymmetry will be a promising way to pursue this goal (Carrion‐Castillo et al., 2020; Francks, 2015). The cross‐sectional design of our studies limits our capacity to make causal inferences between, for example, disorder diagnosis and altered asymmetry. However, most psychiatric disorders are robustly heritable (Geschwind & Flint, 2015), so that future studies may also investigate shared genetic contributions to disorders and variation in brain structural asymmetry (Carrion‐Castillo et al., 2020). Such studies could help to disentangle cause‐effect relations between disorders and brain asymmetry.

## Data Availability

Data sharing is not applicable to this article as no new data were created or analyzed in this study.
